# Aseptic meningitis due to intravenous immunoglobulin therapy (IVIG) in therapy‐refractory pyoderma gangraenosum

**DOI:** 10.1111/ddg.70263

**Published:** 2026-03-25

**Authors:** Katharina A. Kälber, Knut Schäkel, Alexander Enk, Julia K. Winkler

**Affiliations:** ^1^ Universitäts‐Hautklinik Heidelberg Ruprecht‐Karls Universität Heidelberg Heidelberg Deutschland

Dear Editors,

We report on a 33‐year‐old female patient, who first developed extensive livid‐erythematous, partly necrotic and severely painful skin lesions on both thighs and lower legs in 2017. On initial presentation to our dermatology clinic in 2019, extensive ulcerations with necrosis were seen on both thighs (Figure 1). The patient was known to have had ulcerative colitis (UC) since 2010. Initial treatment attempts for UC included azathioprine, ciclosporin A, and infliximab. When the UC did not improve sufficiently, a total colectomy with a pouch insertion was performed in October 2010, followed by an ileostomy. Both clinically and histologically, the ulcerations were compatible with pyoderma gangraenosum (PG); the PARACELSUS score was 11 (Figure 2). PG is a rare neutrophilic dermatosis,^1^ with prevalence rates between 0.3 and 1/100,000 and female overrepresentation. PG can be associated with UC, Crohn's disease, chronic polyarthritis, monoclonal gammopathy, lymphomas, and systemic vasculitis.^2^

**FIGURE 1:**
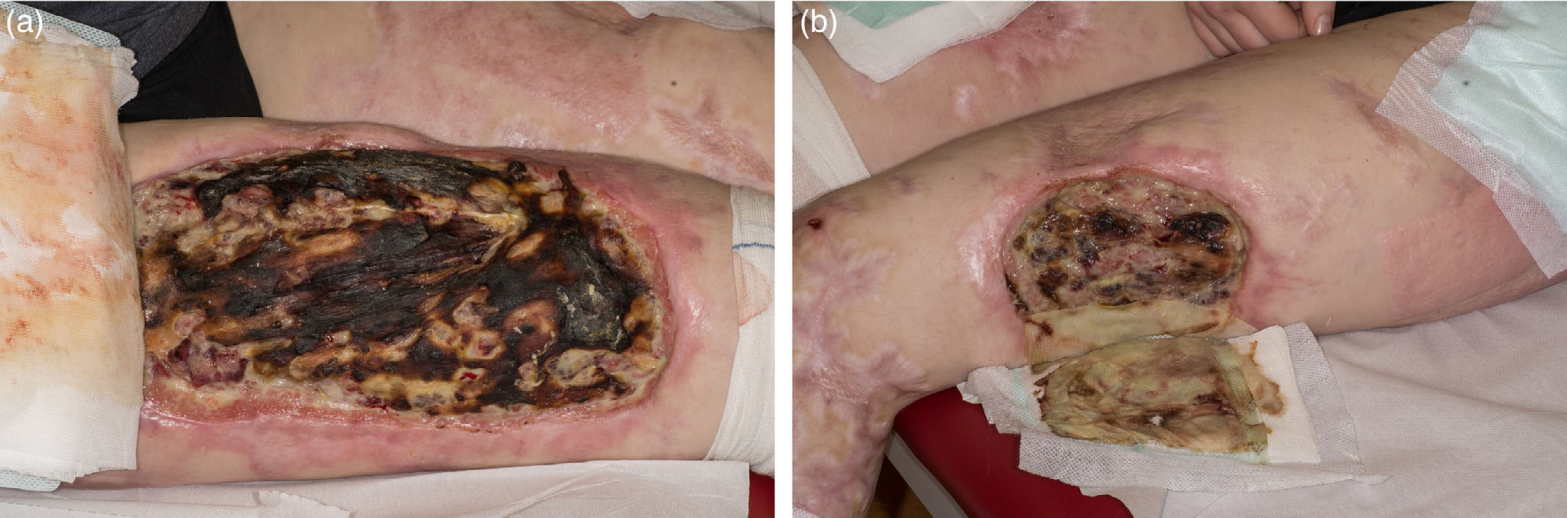
(a) Clinical findings of the initial presentation at our clinic in 2019. Large ulceration measuring approximately 20 x 30 cm on the right thigh with necrosis and muscle exposed. In addition, there are multiple scars from split‐skin grafts. (b) Clinical findings of the initial presentation at our clinic in 2019. On the left thigh, there is an approximately 10 x 18 cm ulceration with necrosis and fibrin layers. In addition, there are scars following split‐skin grafts.

**FIGURE 2:**
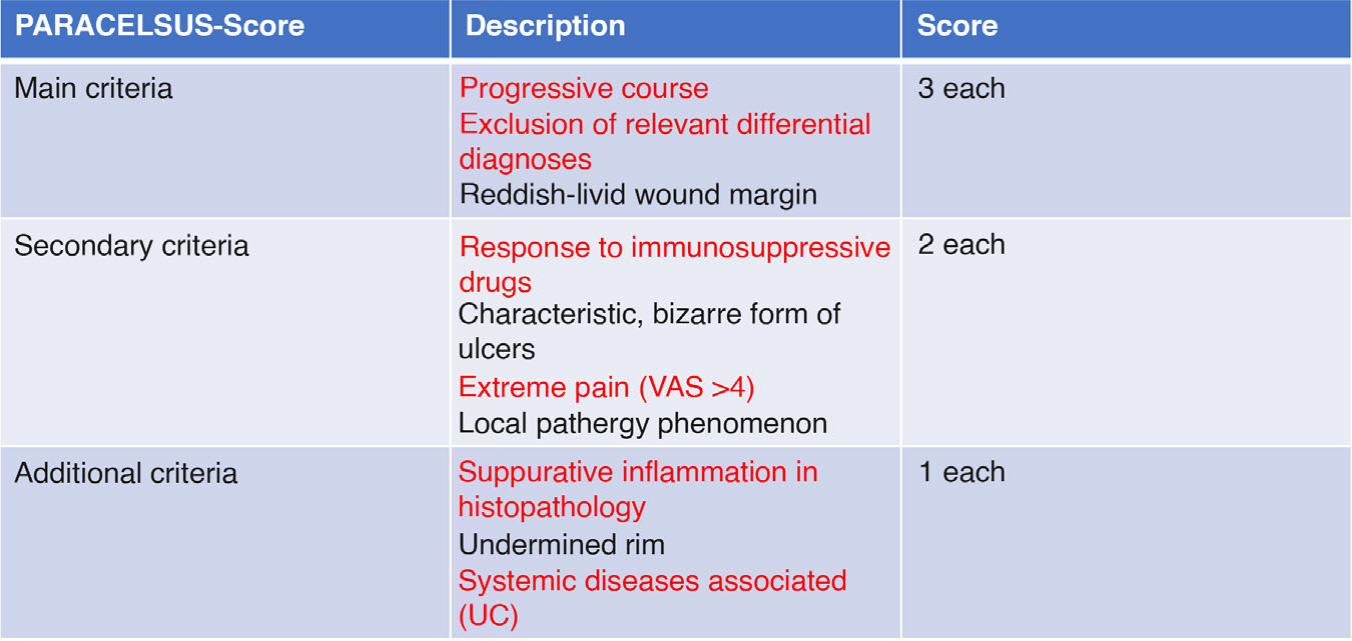
PARACELSUS Score. The criteria that apply to our patient have been highlighted in red. Evaluation: 10 points or more = Pyoderma gangraenosum very likely; < 10 points = Pyoderma gangraenosum very unlikely.

In our patient, dermatitis artefacta was also considered as a differential diagnosis. However, the pronounced sensation of pain, the histopathological findings, the association with UC and the partial response to immunosuppressive drugs argued against a primarily artifactual genesis.

Under azathioprine, systemic prednisolone and adalimumab (Table 1), an initial partial improvement in the findings, but not a sustained remission with complete wound closure, was achieved. A lack of sustained treatment response in PG is possible under immunosuppressive therapies. Prior to the initial presentation in our outpatient clinic, several debridements, vacuum‐assisted closure (VAC), and split‐thickness skin grafts had been performed. These, however, had only led to short‐term improvement, followed by renewed deterioration.

**TABLE 1:** Overview of the systemic treatments between 2010 and 2025.

Period	Therapy	Comment/Reason
07/2010	Azathioprine 150 mg/d	Discontinued due to insufficient effect on ulcerative colitis (UC)
2010	Ciclosporin A^*^	Insufficient improvement of UC
08 – 09/2010	Infliximab 5 mg/kg body weight every 2 weeks	No improvement of UC
11/2017	Prednisolone^*^ + azathioprine^*^	Treatment of pyoderma gangraenosum (PG)
05/2018 – 07/2018	Adalimumab 40 mg every 2 weeks	No sustained clinical improvement
03/2018 – 04/2019	Adalimumab 40 mg + prednisolone shock therapies 20–40 mg/d	No remission
07/2019 – 10/2019	Ciclosporin A^*^ + tacrolimus 8 mg/d	Inpatient admission due to anemia and wound infection
03/2020 – 06/2020	Infliximab 5 mg/kg body weight every 2 weeks	Good clinical effect, but allergic reactions
06/2020 – 09/2020	Infliximab desensitization	Despite premedication flushing/nausea
09/2020 – 11/2020	Omalizumab 300 mg + infliximab 5 mg/kg body weight	Discontinuation due to hypotension and tachycardia after 5th desensitization
01/2021	IVIG high dose 2 g/kg body weight	Aseptic meningitis, anamnestic healing of the PG
01/2025	Prednisolone + subcutaneous administration of beriglobin on own initiative^*^	Insufficient improvement of PG with recurrence since 11/2024
03/2025	Upadacitinib 15 mg/d + mycophenolic acid 1,440 mg/d	Insufficient response
05/2025	Prednisolone 50 mg/d + upadacitinib 15 mg/d + mycophenolic acid 1,440 mg/d	Insufficient response
05/2025	IVIG high dose 2 g/kg body weight	Discontinuation upon recurrence of aseptic meningitis
07/2025	Mycophenolic acid 720 mg/d + tacrolimus 4 mg/d	PG size progressive
08/2025	Mycophenolic acid (monotherapy) 720 mg/d	Deterioration. Tacrolimus due to deterioration in renal function
12/2025	Anakinra 100 mg/d	Improvement of ulceration

* These therapies were externally conducted. No information on the exact dosages could be provided.

Treatment with infliximab was initiated in March 2020. After the first infliximab administration, nausea, flushing symptoms with urticaria, and vomiting occurred but the symptoms improved with glucocorticoids. Prick testing demonstrated type 1 sensitization to infliximab. Desensitization was carried out upon clinical response of the PG to infliximab therapy. However, despite premedication, slow dosing and additional administration of omalizumab, flushing, vomiting, a fear of death, and dyspnoea with tachycardia and hypotension repeatedly occurred. Due to the anaphylaxis, infliximab desensitization had to be discontinued despite its therapeutic efficacy.

Subsequently, treatment with high‐dose intravenous immunoglobulins (IVIG 2 g/kg body weight) was initiated. After the first administration, the patient developed severe headaches with sensitivity to light, vomiting, and fever of up to 39°C. Laboratory chemistry showed slightly elevated infection values. The CSF showed an increased cell count of 42 cells/µl with otherwise unremarkable CSF chemistry. The cytologic examination revealed a lymphocytic cell picture. In addition, there was no evidence of a barrier disorder or intrathecal immunoglobulin synthesis. The patient was admitted to hospital as an emergency case and treated empirically with ceftriaxone and acyclovir. There was no clinical or electroencephalographic (EEG) evidence of encephalitis. PCR for herpes simplex (HSV) and varicella zoster virus (VZV) in the cerebrospinal fluid was negative. Aseptic meningitis after IVIG administration was diagnosed. The immediate temporal relationship between IVIG administration and onset of symptoms within two days, with a rapid improvement after discontinuation of IVIG, was consistent with the diagnosis.

**TABLE 2:** Overview of IVIG side effects and their management. The frequencies were determined according to the following convention: very common (≥ 1/10); common (≥ 1/100, < 1/10); occasional (≥ 1/1,000, < 1/100); rare (≥ 1/10,000, < 1/1,000) and very rare (< 1/10,000).

Side effects	Symptoms	Frequency	Management
Infusion reactions	Headache, nausea, flushing	Very often	Reduce infusion rate, premedication
Headache	Tension or migraine‐like pain	Very often	Hydration, analgesics, reduce infusion rate
Fever / chills	Rise in temperature after infusion	Often	Antipyretics, infusion paused
Hypertension / blood pressure fluctuations	Increase or fluctuation in blood pressure	Often	Monitoring of vital signs, adjust infusion
Tachycardia	Increased heart rate	Often	Observation, reduce infusion rate
Allergic reactions	Urticaria, pruritus, bronchospasm, dyspnoea	Occasionally	Stop infusion, antihistamine, steroids if necessary
Thromboembolic events	Deep vein thrombosis (DVT^*^), pulmonary embolism, stroke, myocardial infarction	Occasional to rare	Minimize risk factors, adequate hydration, thrombosis prophylaxis
Aseptic meningitis	Severe headache, photophobia	Rarely	Discontinue IVIG, analgesics, fluids, exclude infectious causes
Acute renal failure	Increase in creatinine	Rarely	Hydration, change of medication, monitor kidney function
Anaphylaxis	Hypotension, bronchospasm	Very rarely	Emergency treatment: adrenaline, steroids, antihistamines, stop infusion
Transfusion‐related acute lung insufficiency (TRALI)^**^	Hypoxia, pulmonary infiltrate	Very rarely	Immediately stop infusion, intensive care support

* DVT is a blood clot (thrombus) in a deep vein, usually in the legs, which can lead to swelling, pain, redness and pulmonary artery embolism.

** Transfusion‐related acute lung insufficiency (TRALI) is a rare but severe reaction to IVIG and blood transfusions, characterized by sudden shortness of breath, oxygen deprivation and pulmonary edema within up to six hours of transfusion and can be life‐threatening, with damage to the pulmonary capillaries by antibodies in the donor blood.

Treatment of aseptic meningitis after IVIG is based on supportive measures such as analgesics and fluid management.^4^ In most cases, symptoms resolve within one to two weeks without permanent damage.

Aseptic meningitis with high‐dose IVIG therapy is a rare but previously reported side effect^5^ (Table 2). The exact mechanism is not fully understood and different pathomechanisms are being discussed. IVIGs trigger an immune response that promotes the release of cytokines, which can cause inflammation in the central nervous system (CNS). In a case report, the cytokines and chemokines in the CSF of a patient with aseptic meningitis after IVIG and patients with viral meningitis were examined.^6^ Here, increased levels of monocyte chemoattractant protein‐1 (MCP‐1) were found in the cerebrospinal fluid in IVIG‐associated meningitis. It was postulated that the increase in MCP‐1 in the cerebrospinal fluid could lead to the development of aseptic meningitis via the activation of monocytes. In addition, IVIG can form immune complexes that activate the immune system and could thus contribute to the pathogenesis of aseptic meningitis. Some IVIG preparations contain sucrose and/or maltose as stabilizers, which have an osmotic effect and can have a locally toxic or irritating effect on the meninges.^7^

Typical symptoms of aseptic meningitis are headache, fever, neck stiffness and even neurological deficits, occurring within one to seven days after IVIG administration.^8^ A slow infusion rate and sufficient fluid intake are recommended to prevent aseptic meningitis after IVIG; however, no reliable evidence for this exists from prospective studies.^9^

According to the patient's reports, the ulcerations healed completely after the single IVIG administration. As she did not present again in the following three years, this course could not be objectified. In November 2024, the initial diagnosis of chronic thromboembolic pulmonary hypertension (CTEPH) was made in the context of a bilateral pulmonary artery embolism and a pulmonary endarterectomy was performed. At the same time, the patient developed an approximately 35×12 cm recurrence of pyoderma gangraenosum laterally on the right thigh. Inadequate response to systemic glucocorticoids, mycophenolic acid and upadacitinib led to the decision to administer intravenous IVIG again after a critical risk‐benefit assessment. Despite a slow infusion rate, the patient developed nausea, vomiting, severe headaches (VAS 9/10) and a fever of 39.5°C on the third day of IVIG administration. Inpatient treatment for aseptic meningitis was again required. A renewed lumbar puncture was not performed due to the temporal correlation and the anticoagulation following multiple thromboembolic events in the past. The patient received supportive therapy in the form of fluids, potassium substitution for recurrent hypokalemia as well as analgesic and antipyretic therapy. As a result, the symptoms improved rapidly and the patient was discharged in a stabilized condition. Individual epithelial islands subsequently formed in the area of the ulceration on the right thigh, but there was no significant improvement in the findings of the PG.

The interdisciplinary inflammation board recommended a short‐term treatment attempt with highly potent topical glucocorticoids under an occlusive dressing, as the patient had again requested a split‐thickness skin graft procedure.

In addition, further clarification of potential gene mutations that could affect the immune or complement system was also recommended. In the literature, therapeutic approaches with IL‐(12/)23, IL‐36 antibodies, and the IFX‐1 complement C5a inhibitor have been discussed for the treatment of PG.^10^ Several clinical trials are currently being conducted for the treatment of pyoderma gangrenosum. These include a randomized, placebo‐controlled study with the anti‐IL‐36 receptor antibody spesolimab (NCT06624670). Our patient was not treated with an IL‐17 antibody due to her known UC.

Subsequently, the extensive ulceration was covered by plasty. At the same time, treatment with the IL‐1 receptor antagonist anakinra was initiated, which to date has resulted in improved findings.

IVIGs are an established, effective and generally very well tolerated therapy for many autoimmune dermatoses, reducing the need for glucocorticoids and other immunosuppressants.^11^ Most side effects are mild and can be treated symptomatically, but severe side effects such as thromboembolic events, anaphylaxis and, very rarely, aseptic meningitis can also occur.

## CONFLICT OF INTEREST STATEMENT

None.
